# Incidence of dystocia at piglet level in cloprostenol-induced farrowings and associated risk factors

**DOI:** 10.5194/aab-65-97-2022

**Published:** 2022-03-07

**Authors:** Nguyen Hoai Nam, Peerapol Sukon

**Affiliations:** 1 Faculty of Veterinary Medicine, Vietnam National University of Agriculture, Hanoi, Vietnam; 2 Faculty of Veterinary Medicine, Khon Kaen University, Khon Kaen, Thailand; 3 Research Group for Animal Health Technology, Khon Kaen University, Khon Kaen, Thailand

## Abstract

Few studies have investigated risk factors for dystocia in swine, although this birthing abnormality can compromise welfare of both sows and piglets by increasing stillbirth rate and decreasing sow productivity. This study aimed to determine risk factors associated with dystocia at piglet level in cloprostenol-induced farrowings. A dystocia event was recorded when a birth interval exceeded 45 min or when manual extraction was applied. Data were collected from 898 piglets born from 77 Landrace × Yorkshire crossbred sows, which were induced for farrowing on day 114 of gestation. Generalized linear mixed models (GLMMs) were used to evaluate the association between dystocia and parity, gestation length, litter size, relative birth order (RBO (%) = 100 ⋅ birth order/litter size), birth weight, crown rump length, body mass index, ponderal index, piglet's sex, use of oxytocin, and stillbirth. Sows nested in farrowing batches were fitted as random factors in GLMMs. Incidence of dystocia at piglet and farrowing levels was 11.0 % and 75.3 %, respectively. The final multivariate model explained 20.1 % variation of dystocia. RBO had a quadratic effect on dystocia in which incidence of dystocia decreased from RBO ≤ 40 % to RBO = 60 %–70 %, and then increased to the end of parturition. Piglets with birth weight > 1700 g and stillborn piglets had higher odds of dystocia in comparison with piglets with a birth weight of 900–1700 g (OR = 2.63; 95 % CI = 1.66–4.18) and live-born piglets (OR = 2.62; 95 % CI = 1.12–6.15), respectively. This study indicates that dystocia is very common in cloprostenol-induced farrowings and suggests that the last one-third of parturitions is the most important stage to be supervised, and selection for homogenous litters and moderate high birth weight may reduce the rate of dystocia.

## 1 Introduction

During the farrowing process sows are in pain and in distress (Mainau et al., 2016; Navarro and Mainau, 2020). Selection of large litter size in recent decades has resulted in increased farrowing duration (van Dijk et al., 2005; Nam and Sukon, 2020a), which compromises the welfare of both sows and piglets by increasing dystocia rate (Nam and Sukon, 2021b) and stillbirth rate (Mota-Rojas et al., 2012; Nam and Sukon, 2020b, 2021a). In comparison to perinatal stillbirth, dystocia is an unattractive topic because it does not directly relate to the number of piglets born alive as does the stillbirth, resulting in underestimation of this farrowing abnormality.

The incidence of dystocia is usually reported to be very low, that is, 0.25 %–3 % (Jackson, 2004; Cowart, 2007; Parkinson et al., 2018). However, recent studies defining dystocia as an birth interval of more than 30–60 min have provided evidence that dystocia rate at farrowing level varies between 11.0 % and 73.6 % (Boonraungrod et al., 2018; Oliveira et al., 2020). We recently found that in spontaneous farrowings the incidence of dystocia at piglet and farrowing levels was 6.0 % and 47.2 %, respectively (Nam and Sukon, 2021b). Prolonged farrowing duration and birth interval increase stillbirth rate (Baxter et al., 2008, 2009; Nam and Sukon, 2020b) and decrease viability and preweaning survivability (Edwards and Baxter, 2015). This provides evidence of a potentially detrimental effect of dystocia on these criteria. More directly, prenatal survivability decreases with increased farrowing assistance, an indicator of dystocia (Gourley et al., 2020). Moreover, dystocia predisposes sows to increased pain and stress and piglets to a higher risk of manual extraction (Boonraungrod et al., 2018), which subsequently results in an increase in postpartum metritis (Nam, 2020) and culling.

Farrowing induction is used to increase farrowing supervision by forcing sows to farrow during working time (Kirwood, 2015). Prostaglandins and its analogues can be used for farrowing induction (Decaluwé et al., 2014; Boonraungrod et al., 2018). Among them, cloprostenol is the most commonly used drug, and when program starts at day 114 of gestation the stillbirth rate (Gunvaldsen et al., 2007; Gaggini et al., 2013; Otto et al., 2017; Vallet and Miles, 2017; Boonrongraud et al., 2018; Tospitakkul et al., 2019), farrowing duration (Kaoket, 2006; Gaggini et al., 2013; Otto et al., 2017; Boonrongraud et al., 2018), and birth interval (Kaoket, 2006; Boonrongraud et al., 2018) are usually found unaltered.

Several previous studies have assessed effects of various factors including litter size, gestation length, stillbirth, and birth weight on farrowing duration and birth interval (van Rens and van der Lende, 2004; van Dijk et al., 2005; Motsi et al., 2006; Nam and Sukon, 2020a). In a recent report, factors associated with dystocia in spontaneous farrowings were also documented (Nam and Sukon, 2021b). Nevertheless, studies on risk factors for dystocia, particularly in induced farrowings, are still inadequate. Therefore, this study aimed to investigate effects of various factors on dystocia at piglet level in cloprostenol-induced farrowings.

## 2 Materials and methods

### 2.1 Ethics statement

This study was waived from the animal care and use committee of Vietnam National University of Agriculture because it was conducted in a commercial pig farm outside the university where all procedures done on animals have been routinely practiced.

### 2.2 Animals

This observational study was conducted from September 2020 to January 2021 on a farm in Bac Ninh province in the north of Vietnam. Farrowing induction with a 3-week interval has been conducted routinely on this farm since 2017; therefore, sows enrolled in this study might have been induced in this way before. During the study period, in each batch of farrowings about 70–80 Landrace × Yorkshire crossbred sows were induced by the veterinarians of the farm. However, from 5 batches only 77 farrowings born to 898 piglets were fully supervised and therefore included in this study. Investigated sows were between parity 1 and 8 (3.6 ± 2.1). Sows in estrus were inseminated twice with semen of 2 among 12 Duroc boars raised on the farm. Exact paternal information of each litter was not available. Pregnant sows were kept in individual gestation crates. Sows were removed to farrowing crates 5–7 d before the estimated farrowing date. About 2.0–3.0 kg of industrialized feed was daily fed to each sow during gestation. Sows were fed 1.5, 1.0, and 0.5 kg of feed on days 111, 112, and 113 to farrowing. Water was provided ad libitum through a bite nipple drinking system. Sows were vaccinated against classical swine fever, Aujeszky's disease, foot and mouth disease, porcine reproductive and respiratory syndrome, and porcine parvovirus. At day of gestation, cloprostenol sodium was used for farrowing induction (175 µg , HAN-PROST, Hanvet, Vietnam). The injection of cloprostenol sodium was conducted at 07:00 of the working day into the perivulva region of the sows (Kaoket, 2006).

### 2.3 Data collection and definition

Parity number was recorded from the sow card. Gestation length was derived from the information on date of insemination and date of farrowing. Litter size was the sum of the number of live-born, stillborn, and mummified piglets. Birth interval was the interval between the births of two successive piglets. Birth order at which oxytocin (20UI/sow, Oxytocin, Hanvet, Vietnam) was used was recorded. There were no strict rules of oxytocin use, and its use was independent of birth interval. However, oxytocin was not injected before the birth of the fifth piglets. The use of manual extraction was restricted on this farm and was applied when there were signs of reproductive canal obstruction. Relative birth order (RBO) was calculated as following: RBO = (birth order ⋅ 100 / litter size). After being dried with either clothes or hygroscopic flour, all piglets were individually weighted and measured for crown rump length. The measurements lasted for about 35–40 s in each piglet. Body mass index (BMI) and ponderal index (PI) were calculated via the following equations: BMI = (birth weight ( kg ) / crown rump length ( m ) ${}^{2}$ ) and PI = (birth weight ( kg ) / crown rump length ( m ) ${}^{3}$ ), respectively. Colostrum intake was available to all piglets within 1 h of delivery. Piglets were kept in an incubator heated with infrared lamps. Mummified piglets were defined as piglets born dead with full brown/black color of skin. Other dead-born piglets were classified as stillbirths.

This data set was initially used in another study investigating risk factors for intrapartum stillbirths (Nam and Sukon, unpublished data). All stillborn piglets therefore were necropsied for stillbirth classification. However, for the purpose of investigation of risk factors for dystocia, classification results were not presented in this study, and stillbirth as a whole was used as a risk factor for dystocia. A dystocia event was recorded when a birth interval exceeded 45 min or when manual extraction of piglets was applied (Ward et al., 2019; Nam and Sukon, 2021b).

**Table 1:** Comparing some parameters between piglets born with ( n = 99) and without ( n = 799) a dystocia event.

Parameters	Piglet born without dystocia(mean ± SD)	Piglet born with dystocia(mean ± SD)	p
P	3.9 ± 2.1	3.6 ± 2.1	0.238
LS	14.5 ± 3.5	13.4 ± 3.7	0.002
GL	115.1 ± 0.6	115.1 ± 0.7	0.796
BW	1443.7 ± 375.3	1599.5 ± 420.7	< 0.001
CRL	26.5 ± 2.6	27.6 ± 2.8	< 0.001
BMI	17.8 ± 4.6	18.6 ± 5.1	0.085
PI	71.3 ± 13.3	71.9 ± 16.0	0.687
BI	12.0 ± 11.0	93.6 ± 50.7	< 0.001
BO	8.2 ± 4.3	8.1 ± 5.0	0.845
CFD	123.7 ± 93.3	214 ± 128.7	< 0.001

SD: standard deviation; P: parity; LS:  litter size; GL: gestation length; BW: birth weight; CRL: crown rump length; BMI: body mass index; PI: ponderal index; BI: birth interval; BO: birth order; CFD: cumulative farrowing duration; p : probability level.

### 2.4 Statistical analysis

Generalized linear mixed model (GLMM) was used to investigate the effect of different risk factors on dystocia at piglet level. In GLMMs sows nested within farrowing batches were fitted as random factors to take into account potential difference in litters and in batches. Independent risk factors were parity (1–2, 3–4, 5–6, and 7–8), gestation length (114, 115, and 116 d ), litter size ( < 10, 10–15, and > 15), oxytocin use (born before and after the use of oxytocin), sex (female and male), birth weight (400–900, 900–1700, and > 1700 g ), crown rump length (16–27 and > 27 cm ), BMI ( < 16, 16–24, > 24), PI ( < 60, 60–80, > 80), and RBO ( < 40 %, 40 %–60 %, 60 %–70 %, 70 %–90 %, 90 %–95 %, and 95 %–100 %). The partition of independent variables into categorical variables is based on their effect pattern on the outcome. Briefly, the effect of original independent variables was assessed with a chi square to detect the turning points of their effect. Subsequently, the turning points were used as the cutoff values for partitioning these variables into categories (IBM SPSS Statistics for Windows, Version 22.0, Armonk, NY: IBM Corp). The GLMM analysis was conducted with two steps. First, univariate GLMM was used to determine potential risk factors significant at p < 0.1. Second, different combinations of significant independent variables were analyzed with multivariate GLMMs to establish the final model that best explained variation of dystocia at piglet level. Marginal and conditional $R^{2}$ values were derived from measuring the percentage of variance explained by fixed factors and by entire models, respectively. The matching between expected and observed outcomes was tested with Hosmer–Lemeshow goodness-of-fit test. A p value < 0.05 was set as the significance level in the final model. Risk analysis was conducted in RStudio Desktop 1.3.1093 (Boston, MA, RStudio Team: Integrated Development for R).

## 3 Results

The incidence of dystocia at piglet level was 11.0 % ( 99/898 ) and that at litter level was 75.3 % ( 58/77 ); 28, 21, and 9 litters had 1, 2, and more than 2 dystocia events, respectively. The birth interval varied between 0 and 370 min . The stillbirth rate was 5.3 %. Among 77 investigated sows, 11, 42, and 24 sows farrowed at days 114, 115, and 116 of gestation, respectively. Twenty-three and 14 sows needed a single oxytocin injection and manual extraction during farrowing, respectively. Piglets born with dystocia had smaller litter size, heavier birth weight, longer crown rump length, and increased cumulative farrowing duration. Birth interval of dystocic piglets was nearly 8 times longer than that of eutocic piglets. BMI and PI were not different between piglets born with and those born without dystocia ( P > 0.05) (Table 1).

**Table 2:** Univariate analysis of risk factors for dystocia in swine at piglet level ( n = 898).

Covariates	Dystocia rate (%)	OR; 95 %CI; p
RBO < 40 %	12.7 ( 39/306 )	6.27; 1.48–26.53; 0.012
RBO = 40 %–60 %	7.4 ( 14/190 )	3.40; 0.75–15.29; 0.111
RBO = 60 %–70 %	2.3 ( 2/87 )	1
RBO = 70 %–90 %	5.4 ( 10/185 )	2.44; 0.52–11.40; 0.256
RBO = 90 %–95 %	14.3 ( 8/56 )	7.15; 1.46–36.13; 0.015
RBO = 95 %–100 %	35.1 ( 26/74 )	23.44; 5.31–103.53; < 0.001
BW = 900–1700 g	8.1 ( 46/570 )	1
BW = 400–900 g	10.7 ( 8/75 )	1.36; 0.62–3.00; 0.447
BW > 1700 g	17.8 ( 45/253 )	2.46; 1.59–3.83; < 0.001
Born alive	10.4 ( 89/855 )	1
Stillbirth	23.3 ( 10/43 )	2.61; 1.24–5.49; 0.011
P = 7–8	5.2 ( 3/58 )	1
P = 3–4	6.7 ( 6/89 )	1.33; 0.32–5.52; 0.699
P = 5–6	11.0 ( 45/408 )	2.27; 0.68–7.57; 0.181
P = 1	11.7 ( 19/163 )	2.41; 0.69–8.50; 0.168
P = 2	14.4 ( 26/180 )	3.10; 0.90–10.63; 0.073
GL = 114 d	13.1 ( 18/137 )	1
GL = 115 d	10.2 ( 52/510 )	0.75; 0.42–1.34; 0.329
GL = 116 d	11.6 ( 29/251 )	0.86; 0.46–1.63; 0.648
LS > 15	7.7 ( 25/324 )	1
LS < 10	10.9 ( 6/55 )	1.46; 0.57–3.75; 0.427
LS = 10–15	13.1 ( 68/519 )	1.80; 1.11–2.92; 0.016
Born before O ${}^{*}$	10.9 ( 15/138 )	1
Born after O ${}^{*}$	13.9 ( 22/158 )	1.41; 0.83–2.37; 0.203
Female	9.7 ( 43/444 )	1
Male	12.3 ( 56/454 )	1.31; 0.86–2.00; 0.207
CRL = 16–27 cm	7.8 ( 39/499 )	1
CRL > 27 cm	15.0 ( 60/399 )	2.09; 1.36–3.20; < 0.001
BMI < 16	10.0 ( 28/279 )	1
BMI = 16–24	10.5 ( 58/554 )	1.05;0.65–1.69; 0.846
BMI > 24	20.0 ( 13/65 )	2.24; 1.09–4.62; 0.029
PI < 60	10.0 (28.279)	1.23; 0.66–2.28; 0.520
PI = 60–80	13.3 ( 52/392 )	1.68; 0.96–2.94; 0.068
PI > 80	8.4 ( 19/227 )	1

OR: odds ratio; CI: confidence interval; P : parity; LS: litter size; GL: gestation length; BW: birth weight; CRL: crown rump length; O: oxytocin; p : probability level; RBO: relative birth order; BMI: body mass index; PI: ponderal index; ${}^{*}$ inclusion of piglets born from farrowings used exogenous oxytocin before the birth of the last piglets ( n = 23 farrowings).

Univariate analysis of risk factors for dystocia showed that RBO, birth weight, stillbirth, parity, litter size, BMI, PI, and crown rump length were associated with dystocia at piglet level (at p < 0.1) (Table 2). The final multivariate GLMM selected RBO, birth weight, and stillbirth as the most significant factors for dystocia (Table 3). The RBO had a quadratic effect on dystocia in which the incidence of dystocia decreased with the progress of the farrowing bottoming at RBO = 60 %–70 %, then increased to the end of the farrowing. The risk of being born with a dystocia event of the last piglets (RBO = 95 %–100 %) was highest in comparison with other piglets. Piglets with a birth weight of 900–1700 g had a lower risk of being born with a dystocia event when compared with piglets with a birth weight > 1700 g . Stillbirth was associated with increased incidence of dystocia. The final model explained 20.1 % variation of the incidence of dystocia at piglet level. Hosmer–Lemeshow goodness-of-fit test showed good fit between expected and observed outcome ( P > 0.05).

**Table 3:** Multivariate analysis of risk factors for dystocia in swine at piglet level ( n = 898).

Covariates	OR; 95 %CI; p
RBO < 40 %	6.77; 1.59–28.78; 0.010
RBO = 40 %–60 %	3.34; 0.74–15.11; 0.118
RBO = 60 %–70 %	1
RBO = 70 %–90 %	2.38; 0.51–11.19; 0.272
RBO = 90 %–95 %	6.62; 1.33–32.92; 0.021
RBO = 95 %–100 %	22.43; 5.04–99.82; < 0.001
BW = 900–1700 g	1
BW = 400–900 g	1.15; 0.49–2.69; 0.752
BW > 1700 g	2.63; 1.66–4.18; < 0.001
Born alive	1
Stillbirth	2.62; 1.12–6.15; 0.026

OR: odds ratio; CI: confidence interval; p : probability level; RBO: relative birth order; BW: birth weight. Both marginal and conditional $R^{2}$ values were 0.201.

## 4 Discussion

Dystocia in swine is a topic of less importance in comparison with perinatal mortality because it was thought to be uncommon in this species. Documents mentioning dystocia usually report a very low incidence of dystocia in swine, that is, 0.25 %–3 % (Jackson, 2004; Cowart, 2007; Parkinson et al., 2018). It seems that the incidence of dystocia in this animal had been underestimated since recent reports defining dystocia basing on birth interval have shown that this birthing abnormality is not uncommon. Oliveira et al. (2020) reported that about 11.0 %–16.8 % of cloprostenol-induced farrowings experienced at least one dystocia event. This incidence is several times higher in the study by Boonraungrod et al. (2018), where 33.8 %, 50.0 %, and 73.6 % of spontaneous, prostaglandin F2 α (PGF2 α )-induced, and PGF plus carbetocin-induced farrowings needed birthing assistance. In another study, the proportions of sows needed farrowing assistance were 41.7 %, 53.6 %, and 26.7 % in control, single injection, and double injection of cloprostenol, respectively (Tospitakkul et al., 2019). Many previous studies reported that farrowing induction with cloprostenol did not change farrowing duration, birth interval, and stillbirth rate (Kaoket, 2006; Gunvaldsen et al., 2007; Gaggini et al., 2013; Otto et al., 2017; Vallet and Miles, 2017; Boonraungrod et al., 2018). Ward et al. (2019) reported that each farrowing had about 0.8–1.1 dystocia events. In this study, 99 dystocia events were found in 77 farrowings, meaning that each farrowing had about 1.3 dystocia events. Therefore, the incidence of dystocia at piglet level (11.0 %) and at litter level (75.3 %) in this study is expected to be similar to that in the previous study (Ward et al., 2019). Surprisingly, this study is one of few reporting the incidence of dystocia at piglet level and its associated risk factors (Nam and Sukon, 2021b).

The effect of RBO on dystocia in cloprostenol-induced farrowings in the present study is similar to that in spontaneous farrowings in a previous report (Nam and Sukon, 2021b) where incidence of dystocia decreased to RBO of 50 %–60 % and increased thereafter. Similarly, Kovarna et al. (2020) found that birth interval was longest at birth order of 1–4, shortest at birth order of 9–12 and then increased. On the one hand, the quadratic effect of RBO on dystocia/birth interval may be the result of endocrinological change during parturition. Endogenous oxytocin concentration in crated gilts was found to increase during the first hour of parturition and decrease thereafter (Lawrence et al., 1995). Increased oxytocin concentration in the early stage of farrowing stimulates uterine contraction and fetal expulsion resulting in a decrease in dystocia rate in the first half of the parturition. The lowest incidence of dystocia/birth interval reported at different birth order/RBO in different studies may be the result of discrepancy in oxytocin release pattern of investigated sows which was reported by Oliviero et al. (2008). However, it is not known if the lowest dystocia rate/shortest birth interval coincides with highest blood concentration of oxytocin in the farrowing sows. Therefore, further studies exploring the relationship between endogenous/exogenous oxytocin and dystocia are in need in order to establish effective strategies in using oxytocin (if applicable) aiming at reducing dystocia rate. On the other hand, the increased dystocia rate in the second half of parturition may partly be a result of both decreased oxytocin release and uterine fatigue (Mota-Rojas et al., 2007; Motsi et al., 2006).

Interestingly, the present study showed that birth weight was more significant than BMI and PI in explanation of dystocia in swine, although BMI and PI have been reported to be more significant than birth weight and crown rump length concerning stillbirth (Baxter et al., 2008, 2009; Nam and Sukon, 2020b, 2021a). Positive effect of birth weight on dystocia in the present study agrees with the previously reported results (van Dijk et al., 2005). Also, other findings showed that bigger piglets had a higher risk of being hypoxic than smaller ones (Trugillo-Ortega et al., 2007). Bigger piglets have thicker fetal membranes; therefore, it takes them longer to break their membrane before they can travel through the birth canal (van Rens and van der Lende, 2004). Also, in comparison with smaller piglets, heavier piglets have higher friction against reproductive tract. Furthermore, bigger piglets are longer, which also results in their slower movement during the fetal expulsion process. The weight and length of the piglets, however, cannot fully explain their movement speed because the stillborn piglets in this study was smaller (1.12 vs. 1.47 kg ) and had a longer birth interval (36.6 vs. 20.2 min ) in comparison with their live-born littermates. The participation of piglets in the parturition is likely the answer. Only live fetuses can positively participate in the parturition process by actively moving in the reproductive tract, whereas dead fetuses are passively pushed out by uterine and abdominal contraction force (Taverne and van der Weijden, 2008). Keep in mind that bigger piglets are more likely to be born with dystocia but also have a higher survival chance. Therefore, longitudinal studies evaluating the effect of birth interval/dystocia on weaning survival rate of piglets with different birth weights are of interest.

Litter size is negatively associated with birth weight (Nam and Sukon, 2020b, 2021a). Therefore, larger litter size results in lower birth weight, which is the answer to the association between litter size and dystocia in the present study. It is difficult to fully understand the association of parity and dystocia in the present study. Increased dystocia rate in the parity 1 sows can be the result of their narrower birth canals in comparison with sows in other parities and of their small litter size (mean = 13.2). Surprisingly, the highest incidence of dystocia was observed in the parity-2 sows, and this may be attributable to their small litter size (mean = 12.7). However, low dystocia rate in parity 7–8 (mean litter size = 16.5) sows may be the result of selection and culling program rather than the physiological characteristics of reproduction in the high-parity sows. A short range of gestation length (114–116 d ) in the present study prevents any association between gestation length and dystocia from being detected. The use of oxytocin after the birth of the first piglets or 24 h after prostaglandin application shortens the farrowing duration and birth interval; it, however, increases farrowing assistance rate (Muro et al., 2021). In this study its use in the second half of parturition (mean birth order = 6.5) may be only a mild stimulation to fatigued uterine as the dystocia rate did not change.

## 5 Conclusions

RBO, birth weight, and dead-born piglets were the most significant factors for dystocia at piglet level in this studied condition. The present data indicated that dystocia is common in cloprostenol-induced farrowings; therefore, this farrowing abnormality must be considered an important issue during parturition. Dystocia occurs more frequently at the last one-third of parturition when stillbirth usually elevates (Nam and Sukon, 2020b, 2021a). Therefore, this stage is of most importance to be supervised. High-birth-weight piglets are more likely to be born with a dystocia event. Moreover, moderate piglets had the lowest odds of dystocia. Therefore, it is suggested that selection for litters of high homogeneity and nutrition supplementation for increasing litter homogeneity may be approaches to reduce dystocia, which subsequently improves survivability of piglets (Moreira et al., 2019; Yuan et al., 2015) and welfare of the sows.

## Data Availability

The data are available from the corresponding author upon request.
